# Bidirectional regulation of adenosine 5′‐monophosphate–activated protein kinase activity by berberine and metformin in response to changes in ambient glucose concentration

**DOI:** 10.1002/jcb.27312

**Published:** 2018-08-21

**Authors:** Yuanyuan Xiao, Miao Xu, Miriayi Alimujiang, Yuqian Bao, Li Wei, Jun Yin

**Affiliations:** ^1^ Department of Endocrinology and Metabolism Shanghai Jiao Tong University Affiliated Sixth People’s Hospital, Shanghai Clinical Center for Metabolic Diseases, Shanghai Key Laboratory of Diabetes Mellitus, Shanghai Diabetes Institute Shanghai China

**Keywords:** acetyl‐CoA carboxylase (ACC), adenosine monophosphate (AMP)‐activated protein kinase (AMPK), adenosine triphosphate (ATP), berberine, glucose concentration, metformin

## Abstract

Both berberine and metformin are well‐known antihyperglycemic agents for diabetes treatment. Adenosine monophosphate (AMP)‐activated protein kinase (AMPK) activation is often considered as the most important molecular mechanism although the mechanism has been challenged recently. Up to now, when the ambient glucose level changes dynamically, the interaction between AMPK activity and the glucose‐lowering effects of the agents remains largely unknown. To address this issue, HepG2 hepatocytes and C2C12 myotubes were preincubated at normal (5.6 mM), moderate (15 mM), or high (30 mM) glucose concentrations followed by moderate‐glucose incubation plus berberine or metformin treatment. Preincubation at high glucose concentration followed by moderate‐glucose incubation activated the AMPK pathway, but the activation was abolished with berberine or metformin treatment. In contrast, alteration from normal glucose to moderate glucose concentration in the medium suppressed AMPK activity, which was activated by berberine or metformin. Both metformin and berberine decreased the intercellular adenosine triphosphate content, enhanced glucose consumption, and lactate release under all three preincubation glucose concentrations regardless of AMPK activity. In conclusion, AMPK activated by glucose reduction is inhibited by berberine or metformin. The elevation of glucose level led to suppressed AMPK activity, which was activated with the addition of agents. The potent glucose‐lowering effects with minimal hypoglycemia of berberine and metformin may be partially due to their bidirectional regulation of the AMPK signaling pathway. Berberine and metformin promote glucose metabolism via stimulation of glycolysis, which may not be related to AMPK activity.

## 1 INTRODUCTION

Metformin, an insulin‐sensitizing biguanide, is the recommended first‐line oral agent for the treatment of type 2 diabetes.^1^ Berberine, a plant isoquinoline alkaloid, has been used to treat diabetes since 1988.^2^ Our previous studies identified that berberine and metformin may share the same mechanism of action^3^, ^4^ although these two antidiabetic medications have totally different chemical structures.

AMP‐activated protein kinase (AMPK), a heterotrimeric protein serine/threonine (Ser/Thr) kinase, ubiquitously expresses in mammalian tissues, consisting of three subunits (α‐(catalytic), β‐(regulatory), and γ‐(regulatory)) with 2 to 3 genes encoding each subunit.^5^ AMPK acts as a sensor of cellular and whole‐body energy status, playing an important role in the protection of cells during energy crisis. Phosphorylation of AMPKα at Thr172 is used as a biomarker of AMPK activation, which stimulates adenosine triphosphate (ATP)‐producing catabolic pathways and inhibits ATP‐consuming anabolic pathways.^5^, ^6^, ^7^ Regulating lipid metabolism through the phosphorylation of acetyl‐CoA carboxylase (ACC) at serine residues is an important function of AMPK. ACC phosphorylation could inhibit the production of malonyl‐CoA, a substrate for fatty acid synthase and precursor for the synthesis of palmitate. In addition, malonyl‐CoA is a potent inhibitor of mitochondrial carnitine palmitoyltransferase 1 and limits fatty acid β‐oxidation.^8^ Thus, phosphorylation of ACC reduces fatty acid synthesis and induces fatty acid oxidation. It was usually indicated as the activity of the AMPK pathway.

AMPK activation is often considered as the most important molecular mechanism of berberine and metformin although the mechanism has been questioned recently.^3^, ^9^, ^10^ To address this issue, different AMPK activities were induced by the alteration of glucose concentrations in the culture media, and glucose‐lowering effects of the two agents were measured in HepG2 hepatocytes and C2C12 myotubes. This study revealed a bidirectional regulation of AMPK activity by berberine and metformin when ambient glucose level changed. Berberine and metformin could promote glucose consumption via stimulation of glycolysis, which may not be related to AMPK activity.

## 2 MATERIALS AND METHODS

### 2.1 Reagents

Dulbecco modified Eagle medium (DMEM), fetal bovine serum (FBS), penicillin, streptomycin, and other culture reagents were obtained from Gibco Life Technologies (Grand Island, NY). Glucose oxidase was purchased from Shanghai Shensuo Reagents (Shanghai, China). Berberine was obtained from the National Institute for the Control of Pharmaceutical and Biological Products (Beijing, China). Metformin was purchased from Shanghai Sangon Biotechnology Corporation (Shanghai, China). Berberine and metformin were dissolved in ddH_2_O, which was used as the vehicle. All other reagents were, if not indicated otherwise, purchased from Sigma‐Aldrich (St Louis, MO).

### 2.2 Cells

**Figure 1:**
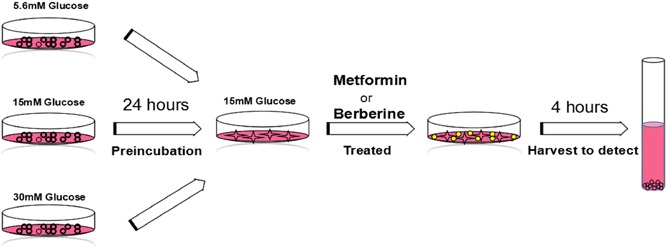
Flowchart of the experiments. The media of different glucose concentration were prepared by adding additional glucose to low‐glucose medium (5.6 mM glucose). The osmotic pressure was adjusted with d‐mannitol (Thermo Fisher Scientific). The HepG2 hepatocytes and C2C12 myotubes were incubated with different glucose concentration for 24 hours. Then, the media were replaced with fresh medium at 15 mM glucose concentration plus berberine or metformin. Four hours later, the cells were collected for Western blot analysis

The human hepatoma cell line HepG2 and mouse skeletal myoblast C2C12 were maintained in a 37°C, 5% CO_2_ incubator and cultured in a growth medium: DMEM supplemented with 10% FBS, 100 U/mL penicillin, and 0.1 mg/mL streptomycin. For differentiation of myotubes, C2C12 myoblasts were seeded into 12‐well plates in DMEM with 10% FBS for 24 hours. Then, the medium was replaced by the differentiation medium: DMEM containing 2% horse serum, 100 U/mL penicillin, and 0.1 mg/mL streptomycin for 6 days. The medium was refreshed every 48 hours. Media of different glucose concentration were prepared by adding additional glucose to low‐glucose medium (5.6 mM glucose). The osmotic pressure was adjusted with d‐mannitol (Thermo Fisher Scientific, Rockford, IL). The cells were incubated with different glucose concentration for 4, 12, or 24 hours, and then treated with berberine or metformin (Figure 1).

### 2.3 Measurement of intracellular ATP content

Intracellular ATP content was detected using an ATP Assay Kit (Beyotime Biotechnology, Shanghai, China). The cells were lysed according to the manufacturer’s instruction. After centrifugation at 12 000*g* for 10 minutes at 4°C, the supernatant was removed and mixed with dilution buffer containing luciferase. The relative light unit was measured by a microplate luminometer according to the manufacturer’s instruction. A fresh standard curve was prepared each time and ATP content was calculated using the curve.

### 2.4 Glucose consumption

The cells were cultured in 96‐well plates and treated with berberine or metformin in FBS‐free DMEM supplemented with 0.25% bovine serum albumin (BSA). The glucose concentration in the medium was determined by the glucose oxidase method. The amount of glucose consumption was calculated by subtracting the glucose concentration of the wells with cells from that of the blank wells.^11^, ^12^

### 2.5 Lactate release

The cells were cultured in 96‐well plates and treated with berberine or metformin in DMEM supplemented with 0.25% BSA. The lactate concentration in the medium was measured with a lactate reagent kit (Shanghai Juchuang Biotechnology Corporation, Shanghai, China).

### 2.6 Western blot analysis

Cells were washed with ice‐cold phosphate‐buffered saline and lysed with lysis buffer (50 mM Tris‐HCL, 150 mM NaCl, 1% Triton X‐100, 1% sodium deoxycholate, 0.1% sodium dodecyl sulfate (SDS), 1 mM sodium orthovanadate, 1 mM sodium fluoride, 1 mM EDTA, 10 μg/mL leupeptin, 1 mM phenylmethanesulfonyl fluoride (PMSF), and phosphatase inhibitor cocktail; pH 7.4). The extracted protein (35 μg) was boiled for 5 minutes, subjected to SDS‐polyacrylamide gel electrophoresis. Then, the separated proteins were transferred onto a nitrocellulose membrane. After blocking with 5% skim milk in the tris‐buffered saline with 0.1% tween‐20 (TBST) buffer for 1 hour, the membrane was incubated with primary antibody at 4°C overnight. Antibodies to AMPK, phospho‐AMPKα (Thr172), acetyl coenzyme A synthetase (ACC), phospho‐ACC (Ser79), and β‐actin were purchased from the Cell Signaling Technology (Beverly, MA). The HRP‐conjugated secondary antibodies (Promega Corporation, Madison, WI) were used with chemiluminescence reagent (Thermo Fisher Scientific, Rockford, IL) for the generation of the light signal; Gel‐Pro Analyzer 4.0 was used to quantify the Western signals.

### 2.7 Statistical analysis

Data are presented as mean ± standard error of mean from individual experiments. Every single experiment was performed at least in triplicate. Student *t* test or one‐way analysis of variance (SPSS 17.0) was used in statistical analysis of the data, with *P* < 0.05 considered significant.

## 3 RESULTS

### 3.1 Alteration of glucose levels changed AMPK and phosphorylation

**Figure 2:**
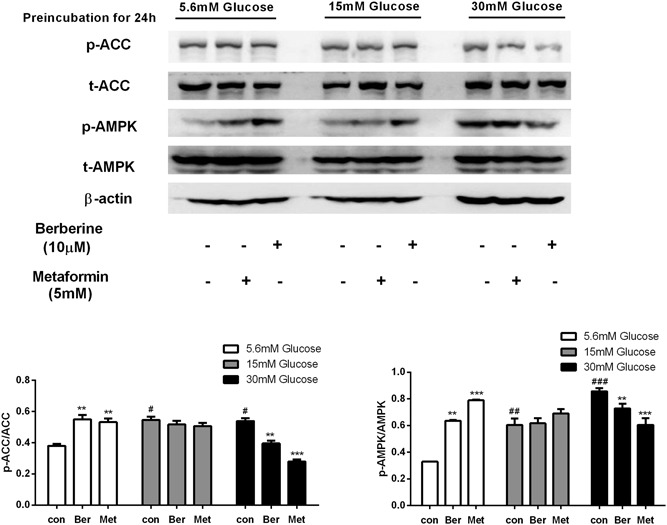
Effects of berberine and metformin on the AMPK pathway in HepG2 hepatocytes preincubated at different glucose concentrations for 24 hours. After preincubation with 5.6, 15, or 30 mM glucose for 24 hours, the HepG2 cells were treated with berberine or metformin at moderate glucose concentration (15 mM) for 4 hours. Then, the cells were collected for Western blot analysis. Data are expressed as mean ± SEM; *P < 0.05, **P < 0.01, and ***P < 0.001 versus corresponding control (without metformin or berberine treatment). ^#^ *P* < 0.05, ^##^ *P* < 0.01, ^###^ *P* < 0.001 versus control of normal glucose concentration (5.6 mM). AMPK, adenosine monophosphate–activated protein kinase; SEM, standard error of mean

**Figure 3:**
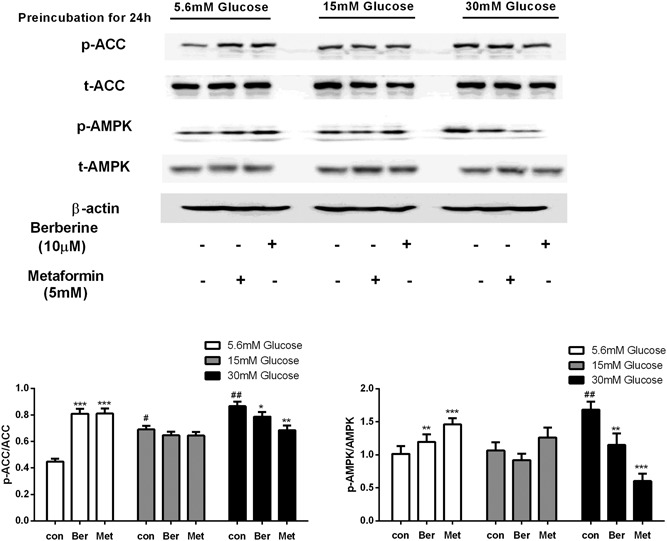
Effects of berberine and metformin on the AMPK pathway in C2C12 myotubes preincubated at different glucose concentrations for 24 hours. After preincubation with 5.6, 15, or 30 mM glucose for 24 hours, the C2C12 cells were treated with berberine or metformin at 15 mM glucose for 4 hours. Then, the cells were collected for Western blot analysis. Data are expressed as mean ± SEM; *P < 0.05, **P < 0.01, and ***P < 0.001 versus corresponding control (without metformin or berberine treatment). ^#^ *P* < 0.05 and ^##^ *P* < 0.01 versus control of normal glucose concentration (5.6 mM). AMPK, adenosine monophosphate–activated protein kinase; SEM, standard error of mean

**Figure 4:**
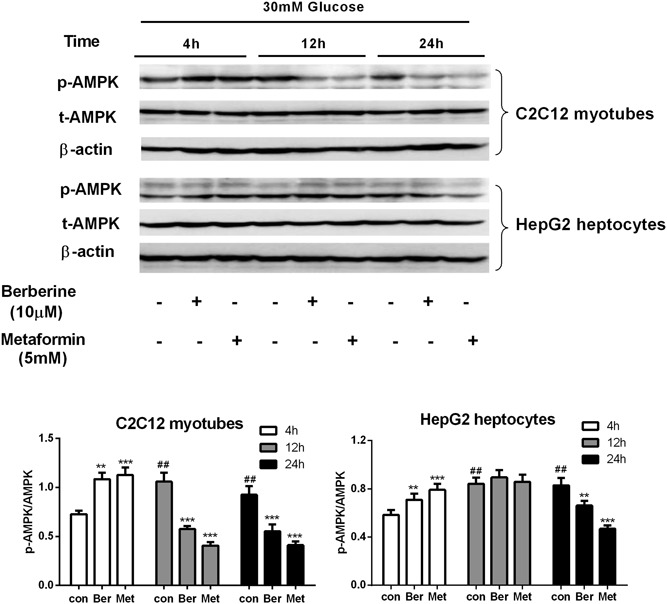
The time‐course effect of preincubation at high glucose concentration on AMPK phosphorylation in vitro. After preincubation with 30 mM glucose for 4, 12, or 24 hours, the HepG2 hepatocytes and C2C12 myotubes were treated with berberine or metformin at 15 mM glucose for 4 hours. Then, the cells were collected for Western blot analysis. Data are expressed as mean ± SEM; **P < 0.01 and ***P < 0.001 versus corresponding control (without metformin or berberine treatment). ^##^ *P* < 0.01 versus control of 4 hours. AMPK, adenosine monophosphate–activated protein kinase; SEM, standard error of mean

Effects of preincubation with different glucose concentrations on the AMPK pathway were detected in HepG2 hepatocytes and C2C12 myotubes as the processing methods shown in Figure 1. ACC, a key enzyme in fatty acid synthesis, was the first protein found to be phosphorylated and inactivated by AMPK. Phosphorylation of ACC was usually indicated as the activity of the AMPK pathway.^13^ The results showed that phosphorylation of AMPK and ACC was stimulated strongly after preincubation at high glucose (30 mM) for 24 hours followed by incubation at moderate glucose (15 mM) for another 4 hours (*P* < 0.05 to *P* < 0.001; Figures 2, 3). On the contrary, phosphorylation of AMPK and ACC was suppressed when glucose concentration in the media increased from 5.6 to 15 mM. To better understand the effect of glucose concentration on AMPK, we measured AMPK phosphorylation after the cells were preincubated with 30 mM glucose at different time points. The AMPK phosphorylation levels of high‐glucose pretreatment for 12 and 24 hours were much higher than that for 4 hours (*P* < 0.01; Figure 4). Our results suggested that glucose reduction activated AMPK. In contrast, glucose elevation diminished AMPK activity.

### 3.2 Berberine and metformin activated the AMPK pathway after glucose concentration ascended

The data above showed that glucose concentration increased from 5.6 to 15 mM greatly inhibited the activity of the AMPK pathway in the cells. However, under the same condition, significant phosphorylation of AMPK and ACC was observed after 10‐μM berberine or 5‐mM metformin was added (Figures 2, 3). Berberine elevated phosphorylation levels of AMPK (Thr172) by 92% and 117% in HepG2 and C2C12 cells, respectively. Similarly, 1.45‐ and 1.8‐fold elevations in phosphorylated ACC (Ser79) were observed after berberine treatment. In addition, metformin caused 2.4‐fold of p‐AMPK and 1.45‐fold of p‐ACC enhancements in HepG2 cells. It also led to 1.4‐fold of p‐AMPK and 1.8‐fold of p‐ACC enhancements in C2C12 cells (*P* < 0.01 to *P* < 0.001; Figures 2, 3).

### 3.3 Berberine and metformin inactivated the AMPK pathway after glucose concentration descended

Although berberine and metformin caused a significant increase of AMPK phosphorylation in HepG2 cells when the glucose level increased from 5.6 to 15 mM (*P* < 0.01 to *P* < 0.001; Figure 2), none of the agents could induce AMPK phosphorylation while the glucose level remained at 15 mM glucose. Moreover, after the glucose level decreased from 30 to 15 mM, AMPK phosphorylation levels in HepG2 cells were reduced by 15% and 29% with berberine or metformin treatment (*P* < 0.01 to *P* < 0.001; Figure 2). A similar effect of berberine and metformin was observed in C2C12 cells, in which AMPK phosphorylation was decreased by 31.6% and 64.2%, respectively (*P* < 0.05 to *P* < 0.001; Figure 3). The suppression of AMPK phosphorylation by berberine and metformin with the high‐glucose preincubation was also tested in a time course. The C2C12 cells were preincubated at high glucose for 4 hours followed by berberine and metformin treatment at moderate glucose, AMPK phosphorylation of the cells was elevated by 56% and 71%, respectively (*P* < 0.01 to *P* < 0.001; Figure 4). However, when the cells were preincubated at high glucose for longer time for example 12 or 24 hours followed by moderate‐glucose incubation, AMPK phosphorylation was stimulated significantly in the absence of the agents. After berberine and metformin were used to treat the cells, the induced AMPK phosphorylation was abolished (*P* < 0.01; Figure 4). The time‐course study was also performed in HepG2 cells, and similar results were obtained. Berberine and metformin induced significant phosphorylation of AMPK after the cells were preincubated at high glucose for 4 hours. However, if the preincubation period extended to 12 or 24 hours, AMPK phosphorylation was stimulated dramatically after the cells were transferred to moderate‐glucose condition, and none of berberine or metformin can further elevate the phosphorylation level of AMPK. In contrast, after high‐glucose preincubation for 24 hours, both berberine and metformin suppressed the AMPK phosphorylation in the HepG2 cells.

### 3.4 Berberine and metformin decreased intracellular ATP content

**Figure 5:**
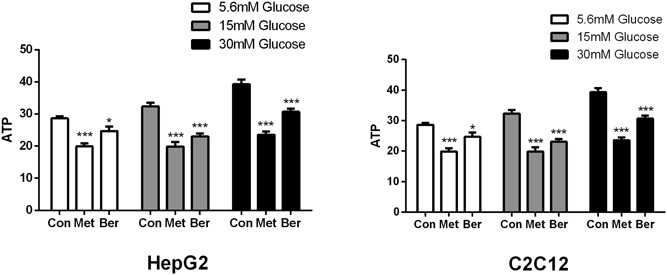
Effects of berberine and metformin on ATP contents in HepG2 hepatocytes and C2C12 myotubes. After preincubation with 5.6, 15, or 30 mM glucose for 24 hours, the HepG2 cells and C2C12 cells were treated with berberine or metformin at 15 mM glucose for 4 hours. Then, the cells were collected for ATP content detection. Data are expressed as mean ± SEM; *P < 0.05, **P < 0.01, and ***P < 0.001 versus corresponding control (without metformin or berberine treatment). ATP, adenosine triphosphate; SEM, standard error of mean

To evaluate the regulatory potential of metformin and berberine on ATP levels, we assessed ATP content in HepG2 and C2C12 cells after treatment with metformin and berberine. The results showed that no matter whether the extracellular glucose concentration changed, both 10‐μM berberine and 5‐mM metformin significantly decreased the intracellular ATP content. Berberine decreased intracellular ATP by 14%, 29%, and 22% in HepG2 (*P* < 0.05 to *P* < 0.001 Figure 5A) and 27%, 16%, and 15% in C2C12 cells (*P* < 0.05 to *P* < 0.001 Figure 5B) after preincubation at different glucose concentrations, respectively. Similarly, in addition, metformin led to 30%, 39%, and 40% reduction of ATP content in HepG2 cells (*P* < 0.05 to *P* < 0.001 Figure 5A), and 20%, 18%, and 17% in C2C12 cells (*P* < 0.05 to *P* < 0.001 Figure 5B).

### 3.5 Berberine and metformin induced glycolysis in HepG2 hepatocytes

**Figure 6:**
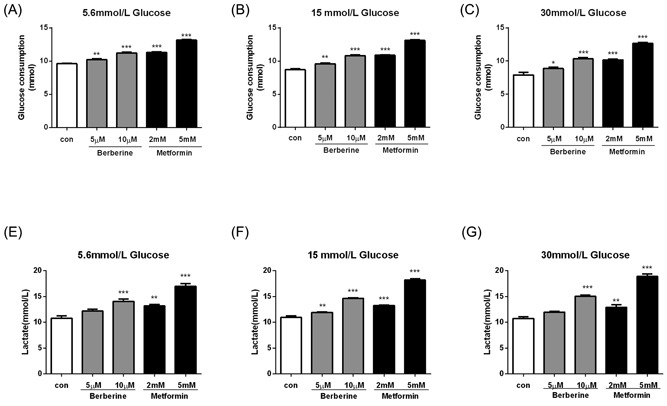
Berberine and metformin induced glycolysis in HepG2 hepatocytes. After preincubation with 5.6 mM (A,D), 15 mM (B,E), or 30 mM (C,F) glucose for 24 hours, the HepG2 cells were treated with berberine or metformin plus moderate glucose concentration (15 mM) overnight. Then, glucose consumption (A‐C) and lactate release (D‐F) were measured. Data are expressed as mean ± SEM; **P < 0.01 and ***P < 0.001 versus corresponding control (without berberine or metformin treatment). SEM, standard error of mean

To further investigate whether alteration of glucose concentration affected the glucose‐lowering effects of berberine and metformin, HepG2 cells were preincubated with glucose concentrations ranged from 5.6 to 30 mM for 24 hours followed by treatment with the agents as mentioned above at glucose 15 mM. After 5.6 mM glucose preincubation, 5 and 10 μM berberine treatment overnight increased glucose consumption by 6.5% and 16.9%, respectively (*P* < 0.001; Figure 6A). The glucose consumption increased by 17.9% and 37% in the presence of 2 and 5 mM metformin (*P* < 0.001; Figure 6A). Similar effects of the agents were observed at the other two glucose concentrations pretreated (*P* < 0.01 to *P* < 0.001; Figure 6B and 6C). To investigate the mechanism of berberine and metformin, lactate release was also measured. Both berberine (5 μM, 10 μM) and metformin (2 mM, 5 mM) increased lactate release in HepG2 cells pretreated at normal (5.6 mM, increased by 12.7% to 30.3%), moderate (15 mM, increased by 8.8% to 34.2%), and high (30 mM, increased by 11.8% to 40.6%) glucose concentrations (*P* < 0.001; Figure 6D‐F). Lactate is the end product of glycolysis. Thereby, these data demonstrated that berberine and metformin were able to induce anaerobic respiration in vitro, and this action was independent of glucose concentrations pretreated.

### 3.6 Berberine and metformin induced glycolysis in C2C12 myotubes

**Figure 7:**
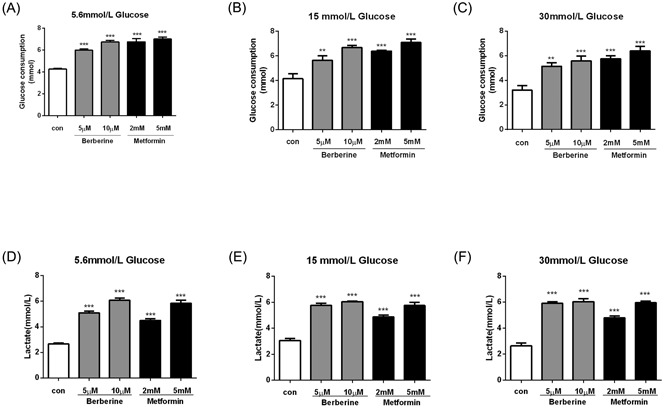
Berberine and metformin induced glycolysis in C2C12 myotubes. After preincubation with 5.6 mM (A,D), 15 mM (B,E), or 30 mM (C,F) glucose for 24 hours, the C2C12 cells were treated with berberine or metformin plus moderate glucose concentration (15 mM) overnight. Then glucose consumption (A‐C) and lactate release (D‐F) were measured. Data are expressed as mean ± SEM; **P < 0.01 and ***P < 0.001 versus corresponding control (without berberine or metformin treatment). SEM, standard error of mean

The glucose‐lowering effects of berberine and metformin were observed in C2C12 myotubes too. After preincubation at 5.6 mM glucose for 24 hours, treatment of 5 μM and 10 μM berberine overnight led to 1.40‐fold and 1.58‐fold elevations in glucose consumption, respectively (*P* < 0.001; Figure 7A). The glucose consumption was increased by 59% and 64% with metformin treatment at 2 mM and 5 mM (*P* < 0.001; Figure 7A). Similar results were obtained in the cells preincubated at 15 and 30 mM glucose concentrations (Figure 7B and C). These two agents also increased lactate release in C2C12 under the same condition (*P* < 0.01 to *P* < 0.001; Figure 7D‐F). These data demonstrated that berberine and metformin were able to induce glycolysis independently of glucose concentrations also pretreated in the myotubes.

## 4 DISCUSSION

In this study, we first found that berberine and metformin played a bidirectional role in regulation of the AMPK signaling pathway. AMPK was inactivated under the condition of glucose excess (preincubation at 5.6 mM glucose for 24 hours followed by incubation at 15 mM glucose); when berberine and metformin were added, the depressed AMPK by glucose excess was stimulated. Conversely, while AMPK had been activated with glucose concentration descending, berberine and metformin suppressed the activated AMPK pathway significantly. For the first time, this study revealed the two‐way adjustment of the AMPK pathway by the agents. That led to precise regulation of glucose metabolism.

The bidirectional regulation of AMPK activity by berberine and metformin may explain why the agents have minimal risk of hypoglycemia in clinical application. Our results suggested that berberine and metformin induced glycolysis and glucose consumption regardless of AMPK activity. When glucose concentration declined, AMPK was activated, whereas berberine and metformin inhibited the activated AMPK. That may prevent hypoglycemia by minimizing AMPK induced energy expenditure. In contrast, when glucose concentration ascended, berberine and metformin could activate the suppressed AMPK pathway and induce glycolysis simultaneously. That may maximize the antihyperglycemic effects of the agents. Thus, this study revealed that berberine and metformin exerted potent glucose‐lowering effects on hyperglycemia, and protective effects against hypoglycemia when glucose level dropped possibly via bidirectional regulation of AMPK activity.

Our current study also aimed to identify whether AMPK activation was associated with glycolysis induced by berberine or metformin. Because Zhou et al^14^ demonstrated that metformin activated the energy sensor AMPK in rat primary hepatocytes in 2001, AMPK was considered as the most important pharmacological target of metformin action. Similarly, several groups including ours reported that berberine was able to activate the AMPK pathway,^15^, ^16^, ^17^, ^18^ which also was believed to be the molecular mechanism of berberine. However, it is controversial whether AMPK activation is essential for the glucose‐lowering effects of berberine or metformin. In this study, on the basis that AMPK was activated after glucose level descended, we found that berberine and metformin did not stimulate the phosphorylation of AMPK and ACC, but inhibited them. Interestingly, even in the absence of AMPK activation, both agents still exhibited potent glucose‐lowering and glycolysis‐inducing effects on human hepatoma cells and mouse myotubes. Other groups also reported that inhibitory effect of metformin on hepatic glucose output or muscular glucose uptake was AMPK independent.^19^, ^20^, ^21^ Our previous study showed that the temporary blockade of AMPK failed to diminish the inducing effects of berberine and metformin on glycolysis.^3^ Furthermore, this study first found the agents even inactivated the AMPK pathway with decline of glucose level and demonstrated that AMPK activation was not related to the antidiabetic effects of berberine and metformin.

In this study, we found that both berberine and metformin significantly decreased intracellular ATP content. Our previous studies revealed that berberine and metformin promoted glycolysis, which was mainly attributed to inhibition of mitochondrial respiration.^3^, ^16^ This study showed that induction of glycolysis and reduction of ATP levels is consistent when the cells were treated with the drugs regardless of glucose concentration alteration. The results further confirm that mitochondrial inhibition rather than AMPK activation plays a central role in the glucose‐lowering effects of berberine and metformin.

In our current study, the HepG2 and C2C12 cells were incubated at different concentration of glucose (normal, moderate and high) followed by the moderate concentration of glucose to imitate different nutrient conditions including nutrient excess and nutrient deficient. AMPK has been identified as a master regulator of cellular energy status and plays a crucial role against energy‐restricted conditions.^22^ Among them, the glucose deprivation is one of the most important factors to regulate the AMPK activity.^23^ Once the depletion of glucose happened, the AMPK will be activated to regulate cellular metabolism by promoting catabolic ATP‐generating pathways, and inhibiting anabolic ATP‐consuming pathways.^6^ In line with previous studies, when the cells were exposed to moderate glucose after incubation at high glucose for 24 hours, the cellular level of AMPK phosphorylation significantly increased, which confirmed the mechanism of glucose deprivation mentioned above. On the other hand, if we treated the cells with normal glucose followed by moderate glucose mimicking nutrient excess, the AMPK activity was suppressed compared with the cells at unchanged moderate glucose concentration. When the cells were pretreated with high glucose for different times, for example, 4, 12, and 24 hours, the decline of glucose level stimulated AMPK phosphorylation was more significant over time. Therefore, it is reasonable to hypothesize that conditions of relative nutrient deficiency, such as blood glucose reduction in hyperglycemia, strict diet control and exercise in the patients of type 2 diabetes, would increase the activity of AMPK. Neutralization of the undesired AMPK activation by berberine or metformin would help to keep the blood glucose in a steady state.

In conclusion, this study revealed a bidirectional role of berberine and metformin in the regulation of AMPK phosphorylation, depending on different nutrient conditions. AMPK, the master energy sensor, was turned on by the agents when glucose level ascended, and turned off when glucose level descended. The dramatic characteristics of berberine and metformin may lead to potent glucose‐lowering efficacy in the absence of hypoglycemia. In addition, berberine and metformin were able to induce glycolysis and glucose consumption, which was not related to the AMPK status. In the future, animal study is considered to further explore the impact of glucose alteration on AMPK activity and the efficacy of antihyperglycemic agents.

## CONFLICTS OF INTEREST

The authors declare that there are no conflicts of interest.
